# The Prevention, Diagnosis, and Treatment of Rapid Oral Health Deterioration (ROHD) among Older Adults

**DOI:** 10.3390/jcm12072559

**Published:** 2023-03-28

**Authors:** Leonardo Marchini, Ronald L. Ettinger

**Affiliations:** 1 Department of Comprehensive Care, Case Western Reserve University School of Dental Medicine, 9601 Chester Ave, Cleveland, OH 44106, USA; 2 Department of Prosthodontics, The University of Iowa College of Dentistry and Dental Clinics, Iowa City, IA 52242, USA

**Keywords:** aged, frail elderly, risk assessment, geriatric dentistry

## Abstract

The world’s population is aging. Older adults are at risk for multiple chronic medical problems as they age. The management of these diseases requires these people to take a variety of medications, which may have undesired side effects. These medical issues can impact oral healthcare and result in a precipitous decline in oral health. A standardized teaching model has been developed to help novice dental practitioners learn how to access and treat oral health problems in older adults. This model is called rapid oral health deterioration (ROHD) risk assessment. The model has four steps for assessment and four categories of risk. This paper describes the components of the ROHD risk assessment, and how it can be used to prevent, diagnose and treat ROHD among older adults.

## 1. Introduction

Population aging is a well-described demographic fact that is reshaping societies across the globe, especially regarding the way healthcare systems are developed and implemented [1]. These systems have been influenced by an increase in chronic diseases that accompanies aging, resulting in older adults using a disproportional share of the healthcare system [2,3]. The high number of chronic illnesses associated with aging, the social repercussions of aging and retirement, as well as the cumulative nature of the most prevalent oral diseases result in an increased risk for rapid oral health deterioration (ROHD) among older adults. The ROHD concept was developed to identify older patients who experienced a decline in general health as they aged, with a concurrent precipitous decline in their oral health [4]. The increased risk of ROHD might help to explain why so many oral health indicators are poorer among older adults when compared to younger cohorts in most countries [5,6,7,8]. This is especially prevalent among the most vulnerable groups, such as institutionalized and homebound older adults [9,10,11,12]. Although the consequences of inadequate oral healthcare can be incapacitating, resulting in localized pain and infection, there are circumstances where more serious oral infections can spread and impact systemic health [13,14].

The evidence-based risk factors for ROHD among older adults can be categorized into three main groups, i.e., systemic health conditions, social aspects, and oral health conditions. Among the most prevalent chronic systemic diseases diagnosed in older adults are arthritis [15], hypertension and diabetes [15,16,17], depression [18,19], neuro-degenerative conditions and dementia [19,20,21], and stroke [15,22]. Older adults usually need to take many medications to treat their multiple chronic diseases, which results in a condition often referred to as polypharmacy [17,23,24].

Aging does not only consist of the biological processes related to senescence because it is influenced by social conditions and how society reacts to aging. These factors will help to determine how a person ages. Often appropriate social support is lacking [25]. The lack of adequate social support also constitutes an important set of risk factors for ROHD. For instance, the inability to afford oral health care and/or the lack of dental insurance after retirement can be an important access-to-care barrier for older adults. In the US, dental insurance is linked to employment. Consequently, it is lost when a person retires, unless he/she can afford to buy private insurance, or is eligible for Medicaid, whose dental benefits vary by state [5,26,27]. Another important barrier is linked to institutionalization since appropriate oral hygiene routines have consistently been reported to be lacking in long-term care institutions [10,12,28].

As the prevalence of tooth loss has declined globally [29,30,31], more and more older adults are able to keep their teeth into old age, which is an important positive achievement of both preventive and restorative dentistry programs. As a consequence, oral health conditions also play an important role in the risk of ROHD. Oral health conditions that increase the risk of ROHD include but are not limited to poor oral hygiene, xerostomia, prosthetic status, heavily restored dentitions, and the presence of gingival recession and root exposure [6,23,32]. These oral health problems also play an important role in treatment planning, as they will impact necessary recall schedules and dental treatment outcomes [33].

Geriatric dental medicine programs have focused on teaching the symbiotic relationship among existing systemic health issues, socio-economic problems, and oral conditions, and how they impact treatment planning for older adults [34,35]. However, the concept of ROHD risk assessment has only been introduced recently [36]. The ROHD risk assessment tool simplifies the teaching of treatment planning, as it can be used by students as a standardized model for caring for their older patients. This risk assessment model is also familiar to students, as it was borrowed and modified from one developed for caries risk assessment [37,38].

When treatment planning using the ROHD risk assessment tool, there are primarily four steps to consider [39]:

(1) Data gathering for evidence-based ROHD risk factors;
(2) Data assessment and prioritization (what matters most?);
(3) ROHD risk categorization;
(4) Identifying viable treatment alternatives.

The data for evidence-based risk factors for ROHD are gathered by taking health histories and medication lists during the patient interview. Other evaluations include an extra-oral and intra-oral examination of the patient, as well as complementary examinations, such as radiographs and other imagery, as well as laboratory examinations, pulp tests, and also mounted dental casts. This process will usually result in a sizeable amount of information, and some items are likely to be more significant for oral disease progression and treatment planning than others. It is important for the practitioner to be able to weigh the importance of specific problems in order to appropriately address them during treatment planning.

**Figure 1:**
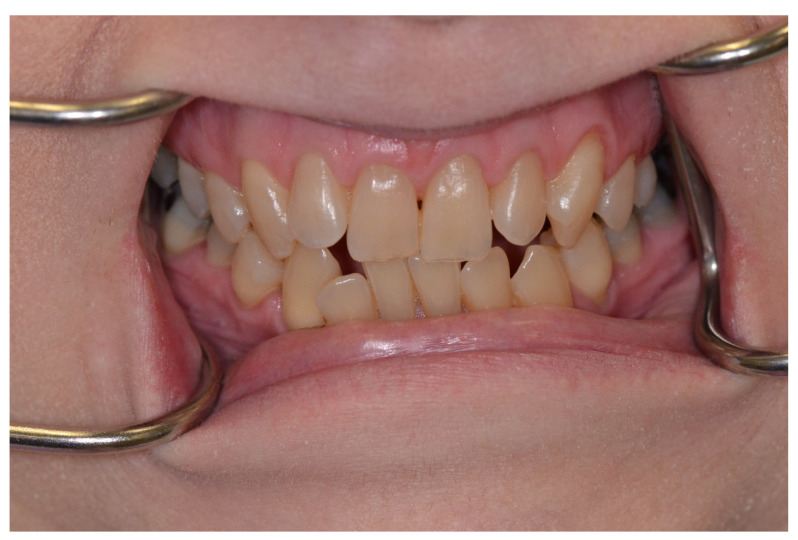
A 62-year-old female has moved to the region recently and seeks treatment. Apart from hypertension, which she controls through diet, medication (thiazide diuretic), and exercise, she has no other systemic diseases. She has all of her dentition, except for the third molar. There is some anterior crowding in the mandible, with minimal bone loss. She has no other important risk factors, and her daily oral hygiene is excellent. This patient represents a person who has no important risk factors, and therefore rapid oral health deterioration is not occurring.

**Figure 2:**
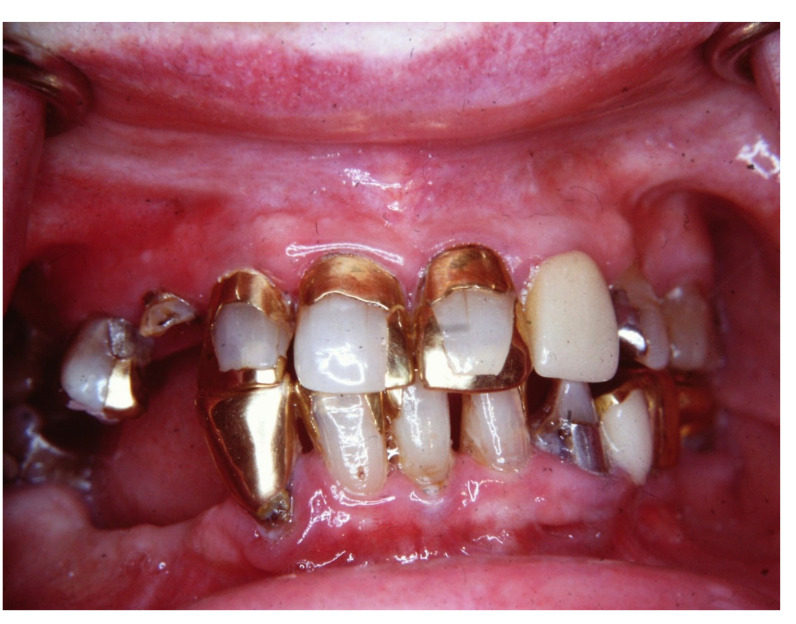
An 82-year-old female is referred for care by her daughter, as the dentist she used to go to has retired. She has a history of allergy to penicillin and sulfa drugs and has been diagnosed with hypertension and coronary artery disease. She also has arthritis in her hips and her hands. She is taking a calcium channel blocker, an ACE inhibitor, and a thiazide diuretic. For her arthritis, she is taking slow-release acetaminophen three times a day. Her chief complaint is that she is beginning to have difficulty eating some of the foods that require more chewing. Her oral examination shows extensive restorative work, which includes several gold inlays. Her periodontal examination revealed no probing depths beyond 3mm and mild bone loss. Although she has a lot of margins at risk, there was no evidence of recurrent caries or root caries. Her treatment will require maintenance care and the addition of a mandibular partial denture. Although the maxillary right lateral incisor is broken down, it was asymptomatic, has no caries or periapical pathology, and she was not concerned with the esthetic correction of this tooth. This patient represents a person who has several important risk factors but does not show any signs of rapid oral health deterioration.

**Figure 3:**
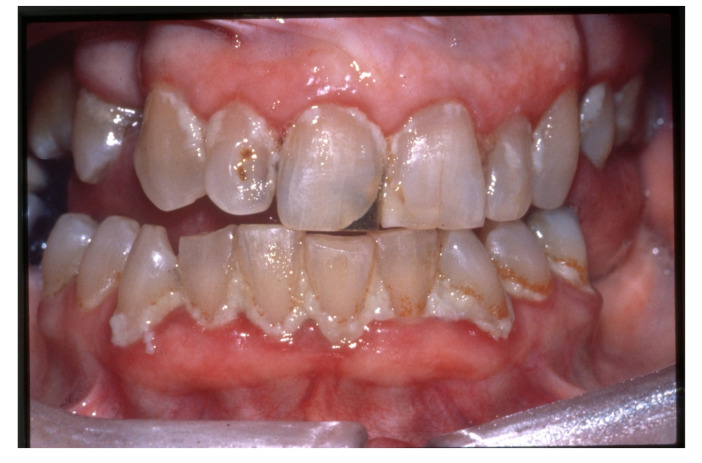
A 75-year-old male was brought to the clinic by his daughter and his wife. The patient has not seen a dentist regularly for the last five years. His wife complains that he is having difficulty eating hard foods. About six months ago, he suffered a cerebral vascular accident that affected the left side of his brain and left him with some weakness in his right side, including aphasia and difficulty walking. Since that time, he also has had difficulty with being able to brush his teeth independently. His other medical problems include hypertension, hypercholesterolemia, arthritis, and type II diabetes. His medications include clopidogrel, metformin, furosemide, potassium, simvastatin, and a calcium channel blocker. An intra-oral examination revealed a dry mouth, heavy plaque, and calculus, especially on the right side of his mouth, accompanied by multiple cervical and coronal carious lesions and marginal gingivitis. His treatment will require significant changes in his daily oral hygiene, which will require his wife to help him. This patient represents a person who has many important risk factors and shows multiple signs that he is developing rapid oral health deterioration.

**Figure 4:**
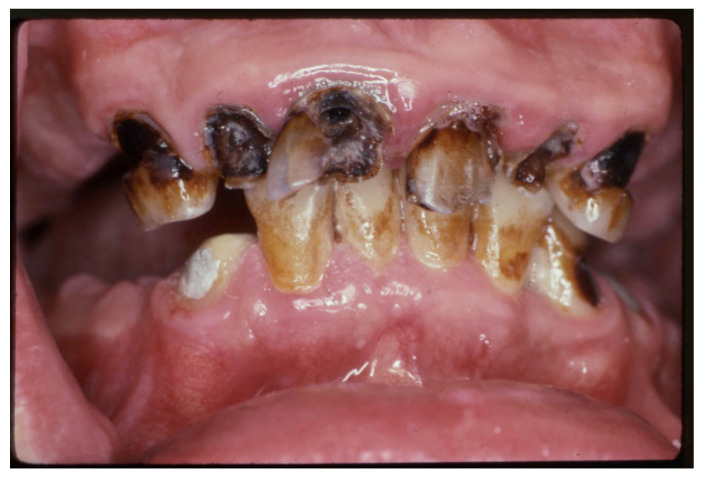
A 65-year-old male is brought for treatment by a social worker as he is in pain. The patient is currently being treated for alcoholism and drug misuse in a hospital setting. He has no family members with whom he is in contact and has been homeless for the last five years. His health history includes smoking a pack of cigarettes a day, hypertension, type II diabetes, osteoporosis, and osteoarthritis. He had not been medicated before hospitalization and is currently receiving naltrexone to help him manage his alcoholism. He is also currently taking an oral antidiabetic, a calcium channel blocker, a thiazide diuretic, and a non-steroidal anti-inflammatory agent. An oral examination revealed multiple carious lesions and root tips, with a sinus tract associated with a mandibular left second premolar root tip, which is the probable cause of his oral pain. The prognosis for his remaining teeth is very poor, and he will need help to find funding for his oral treatment needs. His immediate treatment is the extraction of the infected root tip. This patient represents a person who has many important risk factors, and to whom rapid oral health deterioration has already occurred.

The third step is to categorize the risk of ROHD, which is a function of the risk factors and disease progression, which can be divided into four risk categories [39]:

(1) Risk factors are not present, and there is no ROHD occurring (Figure 1);
(2) Risk factors are present; ROHD has not started (Figure 2);
(3) Risk factors are present; ROHD is happening (Figure 3);
(4) Risk factors are present; ROHD has already happened (Figure 4).

This step-by-step assessment helps the provider to understand the current influence of risk factors on disease progression in order to evaluate what might happen if no intervention takes place, as well as the possible impact of different types of intervention. As a consequence, it helps the practitioner to choose among different treatment options, such as between more preventive or more invasive options. The treatment of older adults can change with time; therefore, the use of ROHD helps the dentist to be aware of changes in the patient’s condition and react appropriately. This step naturally leads to the fourth step, which consists of providing viable treatment alternatives. This includes recommending a specific option and providing the patient and/or caregiver with the rationale for your choice. This will require developing an appropriate communication strategy to address the patient’s needs and to be able to explain it to all involved parties, such as family members, caregivers, or person(s) who have the power of attorney, and other healthcare team members.

This paper aims to provide some examples of how to use the evidence-based risk factors for ROHD to prevent, diagnose, and treat the consequences of ROHD among the growing population of older adults.

## 2. Preventing ROHD among Older Adults

Prevention should be part of any treatment planning strategy for all four ROHD categories. For those patients who are not currently experiencing ROHD, a plan to promote a preventive program should be instituted, so that these patients are less likely to ever experience ROHD. For patients who are currently experiencing ROHD, prevention is key to avoiding further progression of ROHD, such as tooth loss, which is an irreversible consequence of ROHD. When ROHD has already occurred, and there are still some teeth remaining that are healthy enough to be used as abutments for a prosthesis, the long-term survival of these abutments relies on preventive measures [39].

Frequent plaque removal is important to reduce plaque accumulation and therefore control the oral bacterial load. However, if people do not brush their teeth frequently, they have more plaque-related issues, which include gingivitis and caries [40]. Unfortunately, frail older adults are often unable to brush their own teeth. This can occur due to cognitive deficits; some of these patients/residents may only need to be reminded to brush their teeth, and some may need to be supervised while brushing so that they brush appropriately. Nevertheless, some patients/residents will need to have their teeth brushed by their caregivers. Another impediment for patients/residents to be able to brush their own teeth may be physical and not cognitive, such as those who do not have the manual dexterity to brush, or whose vision is so poor that they cannot see what they are doing. Patients/residents in this group may be able to brush their teeth if they have larger toothbrush handles and/or power toothbrushes, while others may still need to be helped by a caregiver. Many techniques have been reported for customizing toothbrush handles to allow older adults with impaired manual dexterity to brush their own teeth [41]. Electric toothbrushes usually have larger handles and have been reported to remove plaque more efficiently than conventional toothbrushes when used by patients/residents or a caregiver [42,43,44].

Increasing exposure to topical fluoride can help reduce caries incidence, arrest existing carious lesions, and prevent new lesions among older adults [45]. A frequently recommended regimen is using 5000 ppm fluoride toothpaste twice a day and having fluoride varnish applied every three to six months. Rising daily with a 0.09% fluoride solution and applying 1.23% fluoride gel every three to six months has also been suggested. This approach, however, presents some challenges, e.g., as the rinse can be easily swallowed by patients with cognitive deficits, and it is difficult for some patients with physical deficits to swish and spit a rinse. In addition, the gel needs to stay in the patient’s mouth for four minutes, which can also be difficult for frail older adults [45].

Silver diamine fluoride (SDF) is a topical fluoride agent that has been used for a long time in many countries to arrest and prevent caries and was introduced to the US market in 2014. The active ingredients in SDF are silver, ammonia, and fluoride [46]. Silver ions inhibit bacterial DNA replication, denaturize bacterial cytoplasmic enzymes, and destroy the cell wall, thus reducing the bacterial load. Fluoride and ammonia improve remineralization and induce the formation of fluorapatite [47].

SDF application is simple and cheap. The technique only requires isolating the tooth with cotton rolls, drying it with an air syringe or a cotton pellet, and then applying SDF using a microbrush for about a minute. The excess can be removed using a cotton pellet [48]. SDF has been reported to be safe [48]. and effective for caries prevention, as well as for arresting caries among frail older adults [49,50]. A contraindication to the use of SDF is an allergy to silver. In addition, SDF stains the carious lesion black, and this needs to be carefully discussed with patients and caregivers before applying the SDF [48].

Xerostomia, or dry mouth, is a very common consequence of polypharmacy, which is frequently observed among older adults [51]. Dry mouth is an important risk factor for ROHD, especially because there is a reduction in the protective mechanisms of saliva, which include its buffer capacity, calcium and phosphate replenishment, and antimicrobial activity [52]. Dentists should always consider some important issues when planning dental treatment for patients with dry mouth or xerostomia. This management includes overall hydration, as patients presenting with dehydration produce less saliva. It also includes the relief of discomfort caused by the absence of saliva, which results in a lack of moisture and lubrication. It is also important to take the necessary measures to prevent dental caries and soft tissue trauma. Saliva also plays an important role in retaining and being able to comfortably wear removable dentures; therefore, the dentist should be aware of the role that saliva plays in denture-wearing [53]. Medications play an important role in the genesis of xerostomia. Consequently, dentists should understand the undesirable side effects of their patient’s medications, and work with other healthcare team members, notably pharmacists and medical prescribers, to evaluate the patient’s medication list in an attempt to reduce their xerostomic effects [53].

After evaluating the patient’s hydration level and evaluating their medications to attempt to reduce their xerostomic effect, the dentist can prescribe saliva stimulants or saliva substitutes to reduce the patient’s discomfort caused by xerostomia and improve their quality of life [39]. Dehydration is a prevalent issue among older adults. However, patients and caregivers are frequently unaware of this condition, as the feeling of thirst declines with age [54]. One way to combat dry mouth, or xerostomia, is to sip liquids regularly. It is not uncommon for older patients/residents to drink juices or liquids rich in sugar as their beverage of choice, thus increasing their risk of developing caries [39]. Consequently, dentists should inform patients and caregivers about the importance of drinking water. Drinking water helps reduce dehydration, as well as the dry mouth sensation, and does not increase caries risk [54].

Some patients may present with drastically reduced salivary secretory capacity, a condition called hyposalivation [53]. For this group of patients, two approaches can be taken. The first one is trying to increase their salivary output by using saliva stimulants, such as lozenges or chewing non-sugared chewing gums. The other approach is to prescribe drugs that induce increased salivary flow, such as bethanechol, anethole trithione, and pilocarpine [52]. Saliva substitutes are not aimed at inducing salivary flow, but instead serve as a replacement for natural saliva. Common forms of saliva substitutes include sprays, oral rinses, and oral gels. By providing moisture and lubrication, saliva substitutes help reduce the sensation of dry mouth and provide some relief for the patients’ discomfort [55]. Dietary changes are another option to reduce the discomfort caused by xerostomia. A less acidic and spicy diet can help to avoid a burning mouth sensation. In addition, there are oral hygiene products specifically formulated for patients with dry mouth, which usually have fewer flavoring agents, such as menthol, and do not use SLS (sodium lauryl sulfate) in their composition [56].

Polypharmacy is a common finding among older adults [17,23,24], and a strong association has been reported between polypharmacy and xerostomia in this age group [51,52]. It has also been reported that medication list reconciliation can help improve patients’ overall health outcomes and reduce adverse drug reactions and healthcare costs [57]. The dentist should work with other healthcare team members, especially pharmacists and medical prescribers, to emphasize the deleterious effects of xerostomia on a patient’s quality of life and evaluate the patient’s medication list in an attempt to reduce the xerostomic effects of the drugs [39].

Xerostomia is an important risk factor for caries among older adults [39]. Therefore, it is important to discuss some strategies which are designed to reduce the risk of developing caries for patients with xerostomia. The dentist should consider customizing these strategies to each individual according to the patient’s ability to manage a preventive therapy and reduce their specific risk factors. The main products that can be used to reduce caries risk are products that induce remineralization of the tooth structure, as well as the use of some other topical antimicrobial agents, such as chlorhexidine [39].

A casein phosphopeptide-stabilized–amorphous calcium phosphate nanocomplex, CPP–ACP, which commercially is called Recaldent, was developed and is the active ingredient of a toothpaste: MI Paste (GC America, Alsip, IL, USA), which can be used as a remineralizing product. This product has been used in clinical trials, which have shown it to be beneficial for patients with xerostomia [58]. It acts by helping to increase the availability of calcium and phosphate in saliva, which induces remineralization [59]. This product can be directly applied to the tooth surface; and, when used at bedtime, can also provide some relief from dry mouth [60]. Regular MI Paste does not contain fluoride, but a newer version, MI Paste Plus (GC America, Alsip, IL, USA), does [61].

Fluoride is another product that can be used to reduce caries risk for patients with xerostomia. An approach that has been used consists of a combination of a high-concentration fluoride toothpaste (5000 ppm) to be used twice a day and the application of a fluoride varnish every 3 months [52]. To maximize the benefit of high-fluoride toothpaste, it is important to explain to patients and caregivers they should not rinse their mouth with water after brushing, and only spit after brushing.

Maintaining an appropriate diet that meets the nutritional requirements is indispensable for the overall health and quality of life among older adults [62]. However, the consumption of sugar is a causative factor for caries, and appropriate control of sugar consumption can help reduce the risk of caries [63,64,65]. Improving diet quality by increasing vegetables and total grains intake has been shown to reduce root surface caries. The consequences of an increased intake of sugar-rich beverages, favored by many older adults, can cause the development of root surface caries [65]. Therefore, it seems important that health practitioners inform patients and caregivers about the importance of having an adequate diet and reducing their consumption of sugar.

## 3. Diagnosing ROHD among Older Adults

When new patients schedule their first appointment in a typical dental office, they need to fill out some forms about their systemic health and dental histories, and personal and insurance information. In order to fill out these forms, patients need to be cognitively intact, literate and have a reasonable understanding of the information being asked. Unfortunately, this assumption is not true for about 59% of American older adults, who have just a basic or below-proficiency level in health literacy [66]. In addition, slower cognitive processing and visual impairments can also make it more difficult for older adults to process the forms [66]. 

The forms completed by patients should be used as a means to begin a dialogue with the patients and/or their caregivers, in order to expand the interview, so that the clinician may be aware of all the other possible risk factors influencing the patient’s oral health. Empathetic listening is very important to fully investigate the social context, the extent of medical conditions, including the medication list, and to better understand the patient’s complaints [33]. 

When assessing a patient’s health history, the clinician should proceed with focused follow-up questions. For instance, if a patient reports a history of *diabetes mellitus type 2*, how does this information influence dental treatment? How is the patient controlling his/her diabetes, and how stable is his/her HbA1c? Did he/she achieve stability through diet and exercise, or is he/she taking an oral antiglycemic medication? Is he/she utilizing insulin, and does he/she have an electronic real-time blood glucose monitor? Unless the patient has had a recent medical appointment or monitors their blood sugar levels on a daily basis, it may be necessary to contact their physician or, if available, to measure their blood glucose level. Prior to treatment, it is also important to investigate if the patient had a meal prior to using their anti-diabetic medication, in order to prevent hypoglycemic episodes. If the patient is stable, then his/her oral health outcomes are less likely to be impacted by the delayed healing or infection related to diabetes. If a surgical procedure is required in an unstable patient with diabetes, it may be necessary to prescribe antibiotics prior to treatment.

Clinical geriatric dental medicine requires data-gathering, in order to problem-solve and make decisions to present patient-centered treatment plans. The factors that influence decision-making and treatment planning for younger adults are relatively simple and are associated with four main factors: first, the patient’s willingness to accept the care; second, the patient being able to schedule time for the delivery of the necessary treatment; third, does the patient have the will and the means to pay for the required care? Finally, does their dentist have the skills, the will, and the resources to carry out the planned care or should the patient be referred?

However, when caring for older adults, the problems tend to become much more complex. Consequently, the oral health care provider should possess more experience and skills in decision-making in order to present the patient with an age-appropriate treatment plan. Age-appropriate care should consider the wide range of modifying factors that older persons are subjected to. These factors can be categorized into socioeconomic factors, systemic health problems, pharmacotherapies, and oral health conditions. Socioeconomic factors include barriers and enablers to accessing dental care, such as transportation issues, lack of dental insurance, and being institutionalized. Systemic health problems include their multimorbidities and the side effects of their medications. Their oral health conditions may reflect the cumulative effects of previous and current dental diseases, as well as any iatrogenic effects caused by previous dental care [67,68].

Planning age-appropriate dental treatments for older adults does not require the development of new technical skills. It does require more in-depth knowledge about the physiological, psychological, and pathological changes associated with aging, as well as its socio-economic consequences. This will allow the dentist to understand how the patient functions in their environment, influenced by their modifying factors so that a dental treatment plan can be developed that fits into their way of living. It is imperative that the benefits of treatment must outweigh any risks or problems related to possible adverse events. Ettinger and Beck have developed a concept of treatment planning for older adults named “rational treatment planning” [69].

As stated earlier, diagnosis and treatment planning for older adults require the gathering of information from and about the patient. A tool to help practitioners process the large amount of information gathered from the patient and make these decisions is known as ROHD risk assessment. By analyzing the ROHD risk assessment, the provider should be able to develop rational treatment plans. 

## 4. Treating ROHD among Older Adults

Treatment plans for older patients can vary depending on the severity of their modifying factors. The treatment can generally be divided into four broad groups, listed below.

### 4.1. Comprehensive Care

To develop an appropriate treatment plan in geriatric dental medicine, the dentist needs to develop skills in problem-solving and decision-making. A treatment plan for older patients should consider all of the modifying factors. These might need to include the patient’s medical problems, the side effects of their medications, their socioeconomic status, and any psychological problems they may have. One must also include the iatrogenic effects of previous dental care, which results in cumulative damage to the dentition [67,68].

Treatment planning for older adults does not require the dentist to learn new technical skills. However, the dentist needs to develop new thought processes in order to understand the more complex modifying factors presented by older adults, as well as how these factors influence treatment. The dentist needs to assess and understand how the patients are functioning in their environments and, consequently, assess how their dental treatment impinges on their lifestyle. Therefore, the dentist should also consider if the benefits of the proposed dental care outweigh the inherent risks of adverse events. Ettinger and Beck have developed a concept of decision-making, which they called “rational treatment planning”. This concept proposes that the dentist needs to evaluate the patient’s modifying factors and develop a treatment plan which is individualized for a patient’s needs; this could be no treatment at all, or the most sophisticated treatment dentistry has available [69]. For instance, if a patient has a limited number of treatable risk factors and can tolerate dental treatment, then comprehensive care is possible [33].

Initially, the dentist would scale and clean the teeth and assess the patient’s ability to maintain oral hygiene independently. If periodontal pockets exist that are deeper than 3mm, non-surgical periodontal therapy, such as deep scaling and curettage, should be considered. If carious lesions are present, excavating the lesions to determine their depth is important. If the lesion is shallow, it may be possible to remove all the caries and restore the tooth. If the lesion is deep, there is data to support partial caries removal, placement of glass ionomers in the deepest areas, and immediate final restoration with a composite, which has been described as the closed sandwich technique [70]. However, if the lesion is very large or very deep, root canal therapy may be required, followed by crowning the tooth. If there are missing teeth, different prosthetic options will need to be discussed with the patient, which might include removable, fixed, or implant options [33,70].

### 4.2. Limited Care

If the patient is frail and cannot tolerate long periods in the dental chair, the treatment may need to be modified. It is essential after scaling and cleaning to establish who is responsible for the patient’s daily oral hygiene routine because the outcome of treatment is dependent on the quality of the oral hygiene [39]. 

Among frail older adults, there is a high prevalence of cognitive impairment, multi-morbidity, polypharmacy, and inadequate ability to maintain daily oral hygiene. This combination puts the patient at risk for aspiration pneumonia if their plaque is not disturbed every five days [71,72,73]. Therefore, it is important to improve oral hygiene routines for frail older adults [74]. For frail older adults who need assistance with daily oral hygiene, the dentist needs to help caregivers with overcoming the barriers in order to provide appropriate care for the patient [75].

If the patient has carious lesions, and it is not possible to use a handpiece, atraumatic restorative techniques (ART) are useful. Traditionally, the ART hand excavates the carious lesions and restores them with glass ionomer [76]. It should also include domiciliary preventive measures, such as the use of high-concentration (5000 ppm) fluoride toothpaste, and 6-month recalls [39]. Another option to treat carious lesions, if esthetics is not an issue, is the use of silver diamine fluoride (SDF) to arrest caries, which is associated with regular 6-month recalls and the reapplication of SDF [50]. All of these patients should have fluoride varnish applied to their remaining teeth during the regular recalls [77]. All prostheses can be considered added risk factors for caries and periodontal disease [78]; therefore, not all missing teeth should be replaced unless they are necessary for chewing and eating, or if the patient requests them for esthetic purposes [79].

### 4.3. Emergency Care (Pain and Infection Control)

The first step in any comprehensive care plan is the treatment of pain and/or infection [80]. For the small group of patients who resist care, it may be the treatment of choice if he or she only seeks care for his/her emergency problem. However, if the patient presents with odontogenic pain and has a dental abscess, the source of the pain needs to be identified and treated. The treatment of the dental abscess will depend upon the size of the lesion and the patient’s will and ability to tolerate root canal therapy. Otherwise, the tooth will need to be extracted. The use of oral antibiotics should be limited to patients where there is an increased risk of spread of the infection [81]. If the clinician diagnoses that pain is from a non-odontogenic source, the cause should be identified and treated appropriately [82]. However, referral of the patient to an appropriate medical or dental specialist may be necessary.

### 4.4. No Treatment

There are circumstances in which a frail older patient presents for dental treatment but has systemic conditions that preclude visits to a dental office, or any invasive procedures in their oral cavity, for example, a patient in the severe stage of Alzheimer’s disease who resists care and also has unstable angina, where stress caused by the patient’s resistance to care could trigger an adverse cardiovascular event. In such a situation, the family and/or caretaker should be encouraged to daily spray chlorhexidine into the patient’s mouth in order to reduce the microbial burden [83].

Occasionally, a patient may seek oral healthcare but, when offered various options for their dental treatment, decides that they do not which to proceed with any of these options. When this happens, the dentist needs to document the refusal in detail in order to avoid any legal consequences. Table 1 summarizes ROHD risk factors, risk categories, and potential treatment modes.

## 5. Conclusions

As older adults age, their risk for multiple chronic medical problems increases. The care and management of these diseases require their physician to prescribe multiple medications, leading to polypharmacy. These medical issues and the side effects of the medications can impact an older adult’s oral health and result in rampant caries and severe periodontal disease. For a novice oral health practitioner, understanding the complexities and interrelation of systemic health issues and dental treatment planning is very difficult. Consequently, to guide novices in decision-making, a standardized teaching model was developed. This concept is named the rapid oral health deterioration (ROHD) risk assessment. There are four steps for assessment in the model, as well as four categories of risk. The four steps are (1) data gathering for evidence-based ROHD risk factors, (2) data assessment and prioritization (what matters most?), (3) ROHD risk categorization, and (4) identifying viable treatment alternatives. The four risk categories are (1) risk factors are not present, and there is no ROHD occurring, (2) risk factors are present, ROHD has not started, (3) risk factors are present, and ROHD is happening, and (4) risk factors are present, and ROHD has already happened. 

This paper described in detail how to use the ROHD teaching model to develop appropriate/rational treatment plans for frail older adults who have both systemic and oral health problems. If a patient has no risk factors and ROHD is not occurring, the dental treatment for this patient will depend upon the time they have available and their financial resources. If a patient has risk factors, but ROHD has not begun, then it is imperative to focus on preventive measures to avoid severe oral health problems. However, if the patient has risk factors and ROHD has started, it is important, as soon as possible, to restore the oral cavity to health within the constraints of the systemic condition, followed by aggressive preventive procedures. Lastly, if the patient has risk factors and ROHD has occurred, the remaining dentition needs to be evaluated to determine which teeth need to be extracted and which key teeth can be maintained to help support a prosthesis if it is required for appropriate function. In certain circumstances, the risk of oral healthcare is too great, and the only treatment is to try to reduce the microbial burden by the use of chlorhexidine.

## Figures and Tables

**Table 1:** Summary of ROHD risk factors, its risk categories, and potential treatment modes.

ROHD risk factors (based on research evidence)
**1. General health conditions** -Cognitive deficits *Alzheimer’s, other dementias* -Functional deficits *Stroke, osteoarthritis, Parkinson’s, etc.* -Sensory loss *Speech, sight, hearing, taste* -Medications *Oral and systemic side-effects, drug interactions* -Manageable chronic diseases *Hypertension, diabetes, osteoporosis, etc.* -Degree of dependence/autonomy *Institutionalization, home care, dependence on caregivers, etc.* -Terminal diseases/palliative care -Life expectancy **2. Social support** -Institutional support -Family/social support -Financial issues *Insurance, Medicaid, Social Security, etc.* -Expectations **3. Oral conditions** -Oral hygiene -Periodontal condition -Number of teeth/restorations -Prosthetic status *Fixed, removable, implants* -Oral lesions *Inflammation, oral cancer* -Stopped seeing the dentist
**Assessment of the Risk for ROHD (based on risk factors and disease progression)**
Risk factors for ROHD are not present. Patient has risk factors for ROHD but is not currently experiencing ROHD. Patient has risk factors for ROHD and is currently experiencing ROHD. ROHD has occurred.
**Treatment alternatives**
Comprehensive Care Limited Care Emergency Care only No treatment

## Data Availability

No new data were created or analyzed in this study. Data sharing is not applicable to this article.
